# Dual-task training of children with neuromotor disorders during robot-assisted gait therapy: prerequisites of patients and influence on leg muscle activity

**DOI:** 10.1186/s12984-018-0426-3

**Published:** 2018-09-17

**Authors:** Sandra Ricklin, Andreas Meyer-Heim, Hubertus J. A. van Hedel

**Affiliations:** 1 Rehabilitation Centre Affoltern am Albis, University Children’s Hospital Zurich, Mühlebergstrasse 104, CH-8910 Affoltern am Albis, Switzerland; 2 Institute of Physiotherapy, Zurich University of Applied Sciences, Winterthur, Switzerland; 3 Children’s Research Centre, University Children’s Hospital Zurich, Steinwiesstrasse 75, CH-8032 Zurich, Switzerland; 4 Physiotherapist in Robotics of Lower Extremity and in the Gait Laboratory Research Associate Paediatric Rehab Research Group Rehabilitation Centre for Children and Adolescents, Mühlebergstrasse 104, CH-8910 Affoltern am Albis, Switzerland

**Keywords:** Adolescents, Surface electromyography, Computer game, Exergame, Lokomat, Driven gait orthosis, Receiver operating characteristics (ROC) analysis

## Abstract

**Background:**

Walking in daily life is complex entailing various prerequisites such as leg strength, trunk stability or cognitive and motor dual task (DT) activities. Conventional physiotherapy can be complemented with robot-assisted gait therapy (RAGT) and exergames to enhance the number of step repetitions, feedback, motivation, and additional simultaneously performed tasks besides walking (e.g., dual-task (DT) activities). Although DT gait training leads to improvements in daily ambulation in adult patient groups, no study has evaluated RAGT with a DT exergame in children with neurological gait disorders. Therefore, we investigated children’s functional and cognitive prerequisites to walk physiologically during RAGT with a DT exergame and analysed the influence of DT on leg muscle activity.

**Methods:**

Children and adolescents (6–18 years) with neurological gait disorders completed RAGT *with* and *without* a DT exergame in this quasi-experimental study. We assessed several measures on the body function and activity domains (according to the International Classification of Functioning, Disability, and Health (ICF)) and determined whether these measures could distinguish well between children who walked physiologically during the DT RAGT or not. We measured leg muscle activity with surface electrodes to identify changes in EMG-amplitudes and –patterns.

**Results:**

Twenty-one children participated (7 females, 6.5–17.3 years, Gross Motor Function Classification System (GMFCS) levels I-IV). Most activity measures distinguished significantly between participants performing the DT exergame physiologically or not with moderate to good sensitivity (0.8 ≤ sensitivity≤1.0) and specificity (0.5 ≤ specificity≤0.9). Body function measures differentiated less well. Despite that the EMG-amplitudes of key stance muscles were significantly lower during DT versus no DT exergaming, the mean activation patterns of all muscles correlated high (ρ > 0.75) between the conditions.

**Conclusion:**

This study is the first that investigated effects of a DT exergame during RAGT in children with neurological gait disorders. Several performance measures could differentiate well between patients who walked with physiological versus compensatory movements while performing the DT exergame. While the DT exergame affected the leg muscle activity amplitudes, it did not largely affect the activity patterns of the muscles.

## Background

Therapy with a driven gait orthosis (DGO) allows a higher intensity of training and facilitates a physiological walking pattern in patients with neurological gait disorders [1, 2]. One major problem of walking in a DGO is, especially in children and adolescents undergoing gait rehabilitation, that patients often remain passive while the DGO moves the legs [3–5]. Due to technological progress, it is possible to combine the therapy in a DGO with a computer game (i.e. an exergame) [6]. Such a game could respond to the leg activity of the patient in the DGO and provide an instant feedback to the child and the therapist [6]. Recent studies in young and adult patients showed that such combinations increased the number of exercise repetitions, the acceptability of therapy, the training duration, the active participation and muscle activation [5, 7–9], and importantly for children, it increased motivation allowing playful learning [5, 7].

While some studies showed that children could improve spatio-temporal gait parameters (e.g., cadence, velocity, and stride length) or walking and standing abilities (e.g., 6 min walk test or Gross Motor Function Measure) through the intensive therapy in the DGO [2, 10, 11], we still observe that children can have difficulties in transferring the improved functions into daily life relevant walking activities. Part of these difficulties might arise from impairments in motor functions such as muscle strength, trunk stability or balance, which are essential for daily life walking. Another factor which we consider important is that in daily life walking is often combined with other motor or cognitive demanding tasks and it is not possible to focus solely on performing leg movements, as can be done in the DGO. To improve the outcome of gait rehabilitation, previous studies recommended to implement dual task (DT) training [12]. For example, patients in an early stage of Parkinson’s disease or chronic stroke could enhance spatio-temporal gait parameters (stride time, cadence, speed, etc.) and reduce their fall risk [13, 14]. Other studies focusing on DT effects included different tasks like walking and a semantic fluency task comparing healthy children, young adults and older adults [15] or the interaction of postural control and visual working memory tasks in children with cerebral palsy [16]. Despite that they already showed effects of a DT (e.g. interference in postural control, in walking distance during given time or a deterioration in the performance of the cognitive tasks) in different populations, we are unaware of studies who investigated effects of a DT combining game playing and walking in a DGO in children and adolescents with neurological disorders.

Based on these findings, we assumed that we could improve effects of robotic gait therapy by introducing a DT exergame. Hence, a DT game was developed where walking in a DGO is combined with upper limb pointing movements. These upper limb movements increase task difficulty, as they distract patients from focusing solely on practising leg movements. We assume that this change in attention might be an approach to train demanding daily life walking skills [6, 12].

So far, no studies have investigated the impact of an attention-demanding motor DT facilitated by an exergame in children with neurological gait disorders. In this first trial, we were interested in the functional and cognitive prerequisites that patients would need to perform such an exergame and how such a DT exergame would influence leg muscle activation. Schuler et al. (2011) [7] already showed that an exergame meant to increase active participation of the child during a DGO therapy indeed increased electromyography (EMG) amplitudes of the leg muscles. However, as a DT exergame could distract the participant from walking, its influence on leg muscle activity and patterns remains unclear.

This study aimed to determine (i) what functional and cognitive characteristics a patient requires to perform such an attention-demanding motor DT with a physiological rather than a compensatory movement pattern, and (ii) whether leg muscle activation amplitudes and patterns change during DT walking. As this game was intended to simulate walking skills during daily life activities, we investigated the ability of several functional and cognitive measures to differentiate between patients who walked with physiological versus compensatory leg movements in a DGO while playing the DT exergame. We hypothesized that walking performance measures could differentiate better between these groups compared to body function measures. Furthermore, we expected that a DT exergame would change leg muscle EMG-amplitudes and activity patterns in children with neurological gait disorders.

## Methods

### Participants

We recruited in- and outpatients in the Rehabilitation Centre for children and adolescents in Affoltern am Albis for this quasi-experimental study in a nine-month time period. As we were unaware of directly comparable studies, we decided not to do a sample size calculation based on assumptions, as this would have resulted in an unreliable outcome. In line with other studies investigating EMG and exergames during walking in a DGO, which included 10 to 24 participants [1, 5, 7, 17], we aimed to recruit 20 participants.

For eligibility, the following inclusion criteria had to be fulfilled: (1) Neurological gait disorders of the central nervous system (congenital or acquired), (2) age of 6 to 18 years, (3) muscle strength, according to the manual muscle test (MMT), of at least M2 of knee and hip flexors/ extensors and M3 in shoulder flexors, abductors and external rotators, (4) DGO experience of at least two therapy sessions, (5) no contraindications for walking in the DGO, (6) a femur length > 23 cm, (7) no severe visual impairments, i.e., have the ability to see the exergame within 2 m distance, (8) capability to follow instructions and communicate fear, discomfort or pain, (9) no surgery or botox injections in lower limbs within the last 3 months.

### Instrumentation

#### Driven gait orthosis

The DGO used in this study was the Lokomat (Hocoma AG, Volketswil, Switzerland). While the participants walked on a treadmill, orthoses guided the leg movements, and a harness facilitated a dynamic body weight unloading. We adjusted the guidance force, bodyweight support as well as the velocity to the individual needs of each participant to facilitate the requirements for physiological walking. We tried to set these configurations as low as possible to minimize support and stimulate activity during walking. However, based on clinical experience from the last 12 years of performing DGO therapies with young patients having neuromotor disorders, we set the guidance force between 80 and 100%. Lower levels are rarely possible, because the young patients have difficulties adopting their regular walking pattern to the induced physiological pattern. Large differences between these two would result in a (safety) stop of the DGO. Nevertheless, also with this rather high guidance force, we often observe that children are still able to walk with some compensatory patterns and muscle activations.

We kept the adjustments constant during the measurement period. During walking, the participant could hold on two parallel bars for better support.

The Lokomat therapy and the measurements were not painful or invasive. To prevent minor adverse events like skin redness or abrasion, muscle or joint pain [18], we carefully adapted the cuffs and the harness and optimized the joint range of motion and the amount of bodyweight unloading.

#### Exergame

**Fig. 1:**
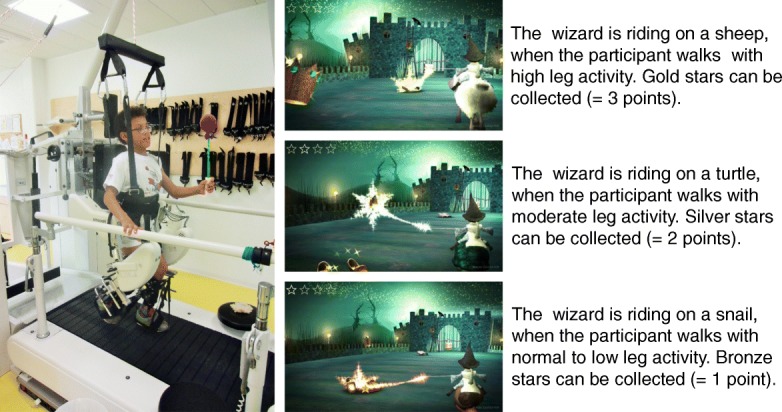
Game and game setting in the DGO. Setting in the Lokomat with a young boy playing the exergame. A short explanation of how the participant’s leg activity changes the virtual avatar animal the wizard is riding on. Thresholds for the transitions between the animals are individually adjusted but calculated in a standardised way

The game “Magic Castle” was developed in collaboration with the Zurich University of the Arts [6]. The participant is represented by a little wizard avatar riding on an animal. This exergame aims to collect points by pointing with the magic wand on sleeping mythical figures (Fig. 1). The participant can wake these characters up by pointing sufficiently long on each character and collect stars (i.e. points). We consider this second task, therefore, a motor-cognitive task, as it requires attention and cognitive flexibility in combination with goal-directed arm movements.

In the exergame, the wizard has to ride through 13 rooms of the castle. Each room represents a game level. The first level was used to explain and try out the game. Our measurement started with the second level. Different rooms or levels showed different sceneries with varying numbers of objects to increase variation and therewith motivation; levels are not increasing in difficulty.

The biofeedback values, measured by sensors in the knee and hip joint of the Lokomat [19] during swing phase, are used by the game to visualize the animal the wizard is riding on. In other words, different levels of the participant’s leg activity result in different animals the wizard is riding on. Please note that completely passive walking was not possible due to the settings of the Lokomat and bodyweight support. Passive walking in the Lokomat would only be possible with extremely high (clinically not used) levels of body weight unloading. In our study, children received body weight support to allow them to accurately and actively extend their knee during stance. If a child would walk passively, it would subside into flexed knees during stance, not showing a proper toe-off because of insufficient extension of the stance leg, and the swing leg would not be able to swing freely. Soon, the Lokomat would measure too high forces against the purposed movement and it would automatically stop (safety stop).

We determined the thresholds for converting between the animals as follows: each individual patient had to walk with the DGO movements, prior to the actual measurements, with normal and with maximal activation levels. These two biofeedback values were used to determine the thresholds between the low and normal and normal and high activity levels. The calculation method was similar to that used by Labruyère et al. [17]. In the exergame, a snail represents low to normal activity (NA). With a little more effort, the snail turns into a turtle (NA + 50% of the range between NA and maximally active), and high activity (NA + 75% of the range between NA and maximally active) changes the turtle into a sheep (see Fig. 1). The thresholds were kept constant for the whole measurement.

During the DT condition, the participant has to point to the characters with a magic wand in the less affected hand to collect stars. We chose the less affected side because it might better reflect the hand used during daily life activities. Less affected was determined according to the clinical diagnosis (e.g. in the case of unilateral cerebral palsy or stroke) or through two items of the handedness test from Chapman et al. [20]. The magic wand is tracked by a colour tracking system from a video camera which is placed on top of the screen [6].

For each aroused figure, the participant gets a bronze, silver or gold star depending on the animal the wizard is riding on (i.e. depending on the level of activation of the legs). Regarding this project, we rated the stars as following: a bronze star: 1 point; a silver star: 2 points; and a gold star: 3 points, see also Fig. 1. Depending on the exergame level, the participant had to collect one to five stars to proceed to the next game level. Therefore, the duration of each level depended to some degree on the performance of each participant.

In the “no DT” condition of the game, the stars were collected automatically, i.e., the participant did not need to point at them, could focus on his leg movements, and held the parallel bars of the Lokomat with two hands. In both conditions, the maximal amount of stars to collect is 34, what results in a maximal possible score of 102 points.

#### Experimental task

**Fig. 2:**
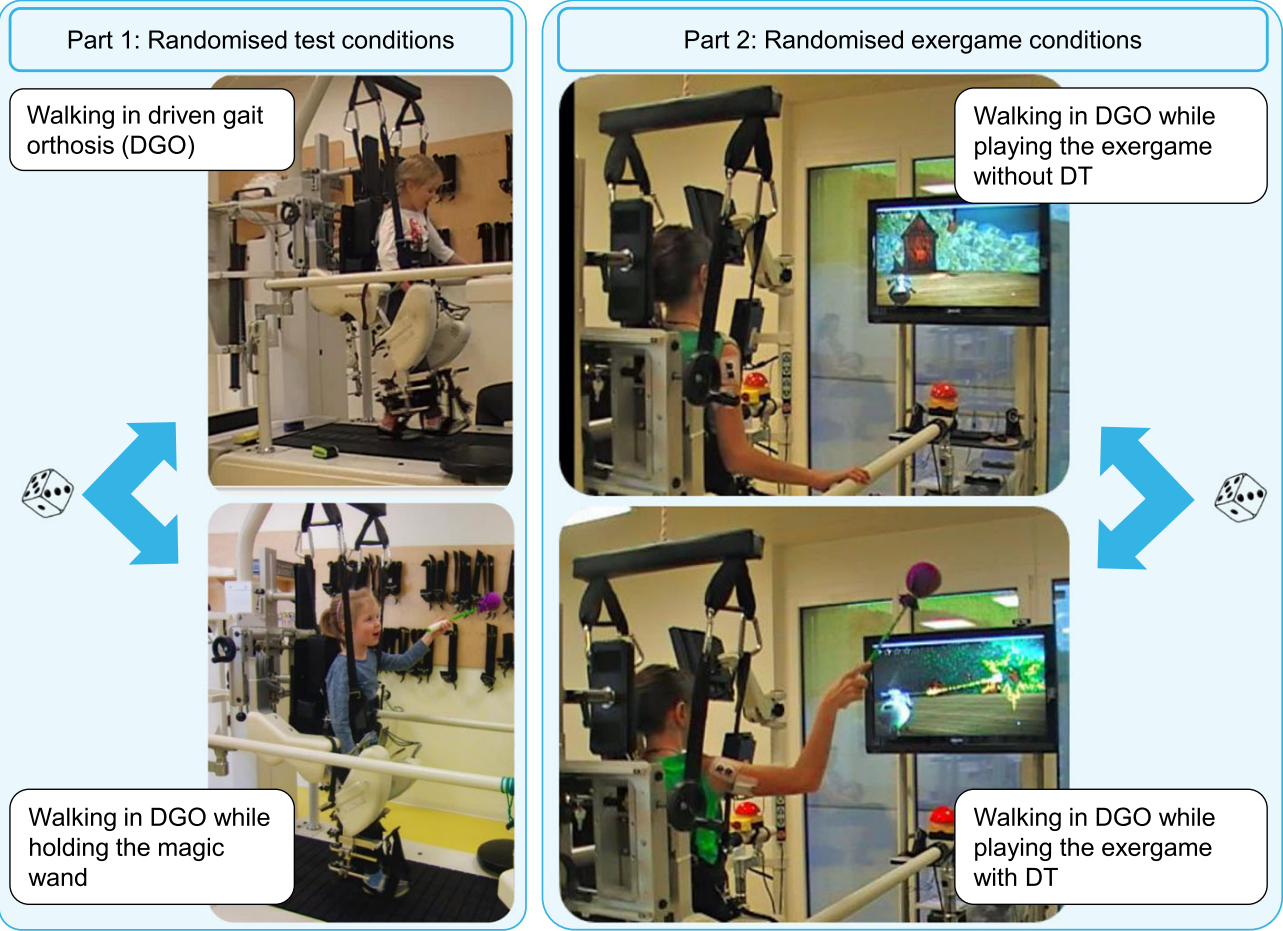
Measurement procedure. Measurement procedure, two test conditions: walking in the DGO with or without reaching movements, two game conditions: walking in the DGO while playing the game with or without reaching movements, within conditions random order. Abbreviations: DGO, Driven Gait Orthosis.

To adjust the bodyweight support, guidance force, and velocity to the walking ability of each participant and to activate blood circulation to prevent a collapse, they walked 4 minutes in the DGO. Then, the DGO was stopped, and the EMG-electrodes were placed and tested. We instructed the participants to walk in the DGO in four randomised conditions (see Fig. 2): with and without reaching movements while playing the game (“game DT” and “game no DT”, each lasting 7 minutes) and two test conditions where the participants walked conventionally (“test DT” and “test no DT”, each lasting 30 s). We included the two test conditions to evaluate the changes in leg muscle activation induced not by altered biomechanics (i.e. lifting one arm) but by the more challenging DT game condition.

We started with the two test conditions (randomized order), followed by the two game conditions (again randomized order). We performed the randomization using https://www.randomizer.org/. The test conditions were always performed first to prevent the occurrence of fatigue or boredom caused by these conditions. The total walking time in the DGO of the children was maximally 35 min without a rest, which corresponds to a usual training duration.

#### Prerequisite measures

According to the International Classification of Functioning Disability and Health (ICF), the domain of body functions and structures can be defined as physiological functions of the body system and anatomical parts of the body like organs, extremities, and their components, while activity is the performance of a task or activity [21]. The participants were characterised by the following performance measures: the Gross Motor Function Classification System (GMFCS I: walks without limitations; V: severely limited self-mobility even with assistive technology, mostly transported in wheelchair) [22], the Manual Ability Classification System (MACS I: handles objects easily and successfully; V: does not handle objects and has severely limited ability to perform even simple actions) [23], the domain mobility of the Functional Independence Measure for Children (WeeFIM mob, maximal score of 35, 5 criteria (three different types of transfers and two types of locomotion) each scored from 7: complete independence; to 1: total assistance) [24] and the Functional Ambulation Classification for basic ambulation skills (FAC, 5: ambulates, independent; 0: non-functional walking) [25]. We routinely assess these measures in our centre. For the ICF body function domain, the 4th version of the Test of Nonverbal Intelligence (TONI4, 60 items in total with dichotomous scoring 1 = correct, 0 = false, assesses two common elements of intelligence like abstract reasoning and problem solving without the confounding effect of motor or linguistic skills) [26] and the Selective Control Assessment of the Lower Extremity (SCALE, scoring of selective movement of each joint in lower extremity, score 2: normal; 0: unable) [27] were evaluated.

Furthermore, a trained Lokomat therapist was asked to rate the participant’s gait pattern during walking in the Lokomat as physiological or compensatory. The same therapist observed and considered the performance of all the participants. She was instructed to focus on the following movements: step symmetry (e.g., symmetrical toe off, heel strike, and step length), compensatory strategies of game playing (e.g., walking with hyperactivity of hip flexion during swing phase, timely accurate knee flexion and extension while walking to enable a proper swing and stance phase), and trunk control (e.g., prominent uni-or bilateral lateral-flexion or flexion of the trunk, not leaning in the harness). At first sight, it might seem redundant to rate, for example, step symmetry, because the Lokomat would guide the legs of the patient in a physiological manner. However, as guidance is limited in the knee and ankle joint (e.g. there is no active drive guiding ankle movements), patients can influence ankle kinematics during toe-off and heel strike, which could affect the step length.

The therapist should also write down the game scores (i.e. bronze = 1 point, silver = 2 points, gold star = 3 points) during the measurements. After each game session, the participants had to rate each game condition regarding motivational aspects (play again yes = 1, no = 0) and difficulty (Visual Analogue Scale (VAS) 1 = big smile = not difficult, 5 = very sad face = very difficult).

We used the TeleMYO DTS system (Noraxon U.S.A, Inc.) with a sampling of 1500 Hz to objectify the leg activity changes during DT and no DT while walking in the DGO. We prepared the skin with an abrasive lotion and a shaver. Self-adhesive Ag/AgCL snap electrodes (Noraxon Dual Electrodes, spacing 2.0 cm) were placed on the *M. quadriceps* vastus medialis obliquus (VMO), M. biceps femoris (BF), M. gastrocnemius caput lateralis (GL) and the *M. tibialis* anterior (TA) of the more affected leg according to the SENIAM guidelines as far as the cuffs of the DGO permitted [28]. We defined the more affected leg according to the results of the MMT (knee and hip flexors/extensors). If there was no difference in the leg muscle strength, we measured the left side. To synchronize the EMG-recordings of the more affected leg with the motor task of the arm, we placed one EMG-electrode on the *M. deltoideus* pars acromialis (DA).

### Data analysis

#### EMG analysis

EMG data was rectified and smoothened with a moving average sliding over the whole measurement (Root-mean-square from a 100 ms window). We used no extra filter. We determined the stance and swing phase manually as well as level changes of the game (no extra activity of the game demanded) using the synchronized video recordings. The steps during level changes were excluded before we exported the EMG data to Matlab (Matlab 7.1, the MathWorks Inc., Natick MA, USA) and transferred to excel sheets.

To analyse EMG-amplitude changes between the DT and no DT condition, we separated the data into stance and swing phase for each muscle and participant. We analysed the mean-EMG amplitudes of the VMO, BF, TA obtained during the swing phase and of the VMO, BF and GL derived during the stance phase in the further analysis, since these muscles are then primary active during the gait cycle phases [29]. The mean EMG-amplitudes were calculated over all steps collected during 5 minutes of exergaming. However, the DA muscle was analysed independently of swing or stance phase.

To compare EMG-patterns, we normalized each step to 100% gait cycle and calculated the average EMG-pattern for each muscle and child with Matlab. We excluded EMG-averages of muscles with signal noise during the whole measurement phase from the analysis.

For the statistical analyses, we included the averaged EMG-amplitudes. However, to differentiate between differences in EMG-amplitudes induced by the cognitive component of the DT and not the differences in EMG-amplitudes induced by changed biomechanics (lifting one arm to point at the objects in the exergame), we also calculated coefficients ($\frac{game\  DT\ }{test\  DT\ }$ versus $\frac{game\  no\  DT\ }{test\  no\  DT\ }$). This procedure is a correction for the biomechanical component.

#### Statistical analysis

To determine how well the body function and performance measures could differentiate between participants walking physiologically or not during the game conditions, we performed a Receiver Operating Characteristic (ROC) analysis. For each measure (ICF activity domain: GMFCS and MACS levels, WeeFIM mobility, and FAC; ICF body function domain: TONI4 and SCALE) we determined cut-off points that distinguished with best sensitivity and specificity between those that walked with physiological versus compensatory leg movements. We calculated the highest Youden Index (YI = sensitivity + specificity – 1) to identify the best combination [30] of the sensitivity and specificity of each value. We considered the discrimination as acceptable (0.7 ≤ Area Under the Curve (AUC) < 0.8), excellent (0.8 ≤ AUC < 0.9) or outstanding (AUC ≥ 0.9) [31].

To determine differences in EMG-amplitudes and EMG-coefficients between the DT and no DT condition, we first evaluated the distribution of the data. We applied the Shapiro-Wilk test and the Levene homogeneity of variance test. According to these results, the mean EMG-amplitudes and coefficients were analysed with the Wilcoxon signed-rank test to compare muscle activity during DT versus no DT in the game condition.

To evaluate the similarity between EMG-patterns between the conditions, we calculated a Spearman’s correlation (ρ) between the muscle patterns recorded during the DT condition and the no DT condition for each muscle and participant. Then, we calculated the mean correlation coefficient over all patients for each muscle activation pattern.

We performed all statistical analyses with SPSS (Version Statistics 24, IBM Corporation, New York, USA).

## Results

We recruited 21 children and adolescents (7 females, 6.5–17.3 years). Characteristics are shown in Table 1.

**Table 1:** Participants‘characteristics

ID	Age (Y)	Diagnosis	GMFCS^b^	MACS^b^	FAC	WeeFIM mob	SCALE mleg	Toni4%	pw DT	pw nDT
1	9.9	Bilateral spastic CP	II	II	4	30	2	48	P	P
2	16.3	Hemiparesis left after cerebral haemorrhage right (23 months ago)	I	II	5	35	6	95	P	P
3	16.2	TBI (20 months ago)	IV	IV	3	18	4	13	C	C
4	8.8	Bilateral spastic CP	II	I	4	33	6	48	P	P
5	13.2	Tetraparesis after Stroke (7 years ago)	I	I	5	33	7	84	P	P
6	16.1	Tetraspastic CP	III	I	5	33	5	37	P	P
7	15.3	TBI (25 months ago), with pre-existing CP	IV	IV	1	19	2	16	C	P
8	12.3	Tetraspastic CP	IV	I	4	28	3	21	C	P
9	17.3	MMC with Chiari II Malformation and corpus colossum agnesia	III	I	4	32	2	32	C	P
10	15.2	Unilateral CP	I	I	5	35	9	66	P	P
11	9.3	Spastic bilateral CP	III	I	4	29	2	50	P	P
12	6.5	Spastic hemiparesis left after HHV-6-Enzephalitis (5 years ago)	II	II	5	34	5	52	P	P
13	9.8	Bilateral spastic CP	III	I	4	26	2	66	C	P
14^a^	6.9	Spastic dystonic tetraparesis after cerebral haemorrhage (4 years ago)	IV	II	1	11	1	19	–	–
15	14.5	Bilateral spastic CP	III	II	4	26	1	26	P	P
16	15.3	Hereditary Neuropathy with cerebellar components	I	I	5	35	7	95	P	P
17	10.3	Spastic tetraperetic CP	I	I	4	32	7	84	P	P
18	10.3	Tetraspastic CP	II	I	5	33	7	55	P	P
19	14.6	Bilateral partly dyston-kinetic CP	II	II	4	29	7	13	P	P
20	13.7	Spastic hemiparesis left after Ependymom WHO Ranking III and haemorrhage (4 years ago)	II	II	4	30	2	14	P	P
21	11.5	Hemiparesis left after cerebral haemorrhage right (1 month ago)	II	II	4	32	2	90	P	P

Abbreviations: *CP* cerebral palsy, *MMC* meningomyelocele, *TBI* traumatic brain injury, *GMFCS* Gross Motor Function Classification System, *MACS* Manual Ability Classification System, *FAC* Functional Ambulation Category, *WeeFIM mob* Functional Independence Measure for children subscale mobility, *SCALE m leg* Selective Control Assessment of the Lower Extremity from the measured leg, *TONI4%* percentile rank score of the Test Of Nonverbal Intelligence 4th version, *pw DT* physiological walking dual-task, *pw nDT* physiological walking during no dual-task, *P* physiological, *C* compensatory

^a^drop out, did not understand the meaning of the game, ^b^GMFCS and MACS levels are rated for all patients for better comparability, although not all were diagnosed with CP

We had to exclude ID14 from further analysis because this participant did not understand the meaning of the game and needed many motivational inputs from the therapist in addition to the standardised instructions.

Patients (*n* = 20) walked with a mean velocity of 1.6 km/h (1.3–1.9 km/h) and a mean body weight unloading of 23.5% (11–45%). The game scores differed significantly (Wilcoxon signed rank test; *p* < 0.001) between game playing with DT (median 25 points, IQR 12–53) and during the no DT condition (median 91 points, IQR 78–98).

During the test condition of regular walking in the DGO, all participants walked physiologically with and without DT. During game playing with DT, five out of 20 participants walked with compensatory movements, whereas during game playing without DT, only one adolescent walked with compensatory movements (see Table 1). Most participants reported that they would play both game conditions again, except from three (ID8, ID12, and ID13). These three participants rated the game with DT as “very difficult” and ID 8 and 13 also walked with compensatory movements during DT.

**Fig. 3:**
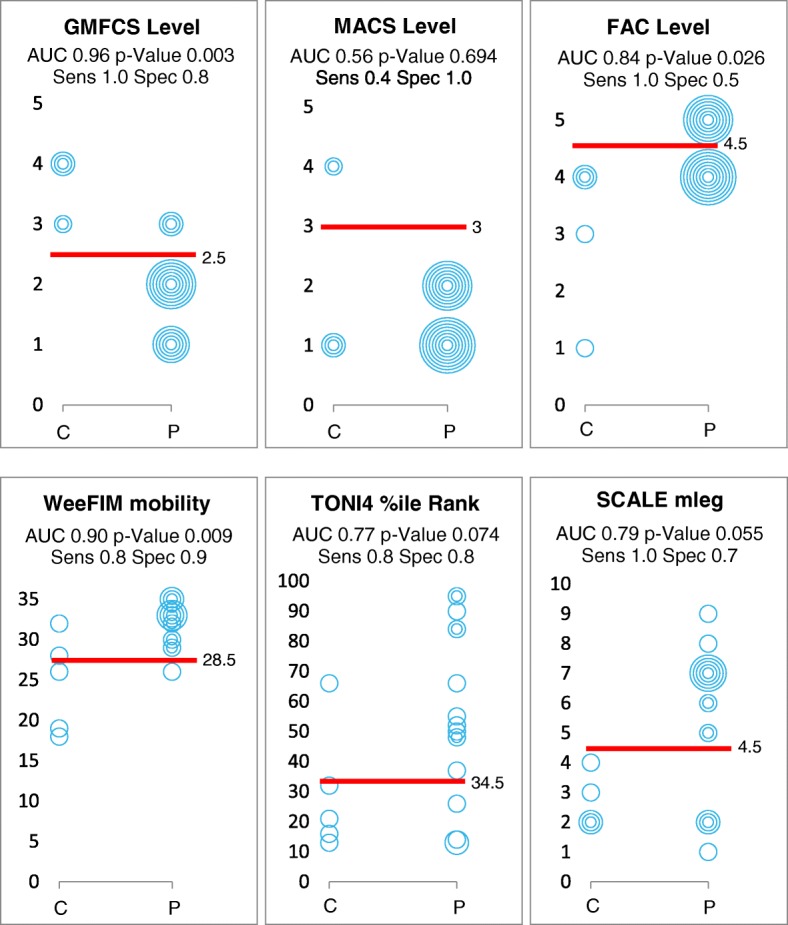
Scatterplots of the prerequisite measures and the therapist rating. Scatterplots combining walking performance (physiological = p, compensatory = c) and the functional and cognitive prerequisites. Abbreviations: GMFCS, Gross Motor Function Classification Scale; MACS, Manual Ability Classification System; FAC, Functional Ambulation Classification; WeeFIM, Functional Independence Measure for children; TONI4%ile rank, percentile rank score of the Fourth version of the Test of Nonverbal Intelligence; SCALE mleg, Selective Control Ability of the Lower Extremity from the measured leg; AUC, area under the curve; sens, sensitivity; spec, specificity.

Scatterplots in Fig. 3 show the cut-off values of the GMFCS level, MACS level, FAC, mobility subscale of the WeeFIM, TONI4, and SCALE that distinguished best between participants walking physiologically (p) or with compensatory movements (c) during the DT game condition in the DGO. Cut-off values, as well as the corresponding AUC, *p*-values, sensitivity and specificity, are displayed. Best results (i.e. highest Youden Index) are achieved in the ICF domain activity (GMFCS level, WeeFIM and FAC). On the ICF domain body function, measures distinguished less well (see Fig. 3). The cut-off value 3 of the MACS level shows the worst results in the Youden Index and ROC. Cut-off values for the game condition without DT were lower for the most measures (GMFCS level: cut-off 3.5, sensitivity 0.90, specificity 1.00, AUC 0.95, *p* = 0.140; WeeFIM mobility subscale: cut-off 22.5, sensitivity 0.95, specificity 1.00, AUC 1.00, *p* = 0.099; FAC cut-off 3.5, sensitivity 0.95, specificity 1.00, AUC 0.95, *p* = 0.14 and percentile rank score of the TONI4: cut-off 13.5, sensitivity 0.95, specificity 1.00, AUC 0.97, *p* = 0.119). They remained similar for the SCALE of the measured leg (cut-off 4.5, sensitivity 0.58, specificity 1.00, AUC 0.58, *p* = 0.800) and the MACS level (cut-off 3, sensitivity 0.95, specificity 1.00, AUC 0.97, *p* = 0.119).

Results of the Wilcoxon signed-rank tests to check for changes of mean EMG-amplitudes and coefficients are shown in Table 2.

**Table 2:** EMG-amplitudes/ - coefficients and results of Wilcoxon signed rank test

Muscles	gait cycle	EMG-amplitudes [μV] game DT median, IQR 25–75	EMG-amplitudes [μV] game nDT median, IQR 25–75	N of analysed pairs	*p*-values of Wilcoxon ^*^	EMG-coeff. DT conditions median; IQR 25–75	EMG-coeff. nDT conditions median; IQR 25–75	N of analysed pairs	*p*-values of Wilcoxon ^*^
VMO	stance	30.6 (2.0–161.6)	34.7 (2.0–246.6)	19	0.001	1.0 (0.8–1.1)	1.1 (0.9–1.3)	15	0.003
	swing	10.4 (2.9–137.4)	10.6 (2.2–137.4)	19	0.212	1.2 (0.7–1.4)	1.0 (0.7–1.4)	15	0.281
BF	stance	15.0 (6.5–166.4)	15.6 (4.3–170.9)	20	0.313	1.1 (0.9–1.4)	1.3 (1.0–2.1)	16	0.023
	swing	11.2 (4.9–59.7)	11.3 (4.3–71.7)	20	0.218	1.5 (1.2–1.8)	1.4 (1.0–2.3)	16	0.642
TA	swing	14.8 (1.6–36.0)	15.3 (1.6–60.1)	18	0.064	1.1 (0.7–2.3)	1.0 (0.9–2.3)	14	0.594
GL	stance	12.6 (4.8–597)	14.7 (2.0–28.9)	18	0.122	1.2 (0.9–1.4)	1.5 (1.1–1.8)	14	0.009

^*^inconsistent normal distribution of data, therefore nonparametric analysis

Abbreviations: *DT* dual task, *nDT* no dual taks, *VMO* vastus medialis obliquus, *BF* biceps femoris, *TA* tibialis anterior, *GL* gastrocnemius lateralis, *DA* deltoideus acromialis, *EMG-coeff*., electromyography coefficients, *IQR* interquartile range

When comparing both game conditions with each other, the VMO EMG-amplitude is significantly lower during stance and DA EMG-amplitude significantly higher during game playing with a DT (DA EMG-amplitude DT: 61.6 μV (14.0–146.4 μV); no DT: 13.7 μV (4.1–60.3 μV); *p* = 0.001). However, when we corrected for the biomechanical component of lifting one arm and we analysed the coefficients, all mean EMG-amplitudes of the key muscles during stance phase are reduced significantly during the game condition with DT versus without DT. The missing values from TA and GL occurred because two children were not allowed to walk without orthoses. Therefore, the placement of the EMG was not possible. Additionally, we excluded one value of the VMO from the analysis, because of signal noise caused by friction of a Lokomat-cuff. Since we started to measure the test condition after the first four participants were measured, we could not calculate the coefficients for them.

Muscle activation patterns correlated quite well when comparing the two game conditions. The mean correlation coefficients were 0.81 for VMO activity pattern (15 out of 19 ρ > 0.75), 0.79 for TA (13 out of 18 ρ > 0.75), 0.84 for BF (16 out of 20 ρ > 0.75) and 0.76 for GL (14 out of 18 ρ > 0.75).

## Discussion

We induced a DT through this exergame to create a multitasking and demanding robot-assisted gait therapy to simulate prerequisites needed for daily walking situations. In contrast to regular robotic gait therapy, this DT exergame made it difficult for the patients to focus continuously on the legs. This kind of walking is in line with normal ambulation in daily life, where it is rarely possible to focus just on leg movements because of several disruptive factors [32]. Another indication that this DT exergame is harder to play than standard gaming during walking is the significant poorer game score during DT exergaming, as the game scores are associated both with accurate arm movements and appropriate leg activations.

We had formulated two hypotheses a priori. We can confirm our first hypothesis, namely that walking performance measures could distinguish better between patients who performed the DT exergame with physiological versus compensatory movements than body function measures. Especially participants with GMFCS level I or II, a WeeFIM mobility score exceeding 28.5, or an FAC greater than 4.5 showed a physiological walking pattern while game playing with a DT. These results could indicate that their multitasking ability might already be better. While we could verify this for the GMFCS, as this correlated moderately with the game score during the DT condition (ρ = − 0.46, *p* = 0.044, *n* = 20), but we found no significant correlations with the FAC or WeeFIM mobility scores. Apparently, also these children with high motor abilities were challenged by the DT condition, also because the game scores differed largely between the conditions. Additionally, we would like to mention that in our participants, the MACS level did not seem to be a crucial factor for the ability to play the DT game condition with a physiological walking pattern. On the one hand, the children played the game with their less affected hand. On the other hand, because of the inclusion criteria of at least M3 (according to MMT), just two were rated with a MACS level IV and all others with II-I.

Interestingly, in the paper of Laburyère et al. [17], game scores could distinguish well between children with neurological gait disorders who performed desired versus non-desired movements during game playing while walking in a DGO (AUC 0.93, *p* < 0.01). In contrast, the scores of our new exergame could not distinguish that good between a physiological or compensatory walking performance during DGO game playing (cut-off score for physiological walking during DT game 31.5, AUC 0.6, *p* = 0.5, sensitivity 0.47, specificity 1. Cut-off score for physiological walking during no DT game score 76, AUC 0.84, *p* = 0.26, sensitivity 0.84, specificity 1). This could be due to the different therapeutic targets these exergames have and, as a consequence, they demand different patient prerequisites. The exergame investigated by Labruyère et al. [17] requires a higher selective control in the legs while solely focusing on walking whereas the exergame in this study requires a shared attention for selective leg movements and a target-oriented arm task.

We can partly confirm our second hypothesis, namely that a DT exergame played while robot-assisted walking would change leg muscle EMG-amplitudes in children and adolescents with neurological gait disorders. We found significantly lower EMG-amplitudes of all key muscles during stance in the DT game condition compared to the no DT condition, which leads to the assumption that there are still some effects of a DT in all our participants. We suggest that this significant reduction can be explained by the neglect of active leg extension during stance while focusing on the game and the arm tasks. The patients might still have limited multitasking abilities and they are not able to perform both tasks without any effect in one or both tasks (i.e. EMG-amplitudes or game points). In daily walking, a reduction in muscle activity during stance could lead to a reduced stability and an increased risk of falling. Since robot-assisted walking provides a safe environment where falling cannot happen, it may be possible to train the multitasking ability of walking at an earlier stage of rehabilitation. Yogev-Seligman et al. 2008 [12] mentioned that interventions which train the walking ability while performing another task could be successful at improving gait and reducing the negative consequences of a secondary task during gait (e.g., impact on functional abilities and fall risks).

The results regarding the EMG-amplitude reduction might give the impression that leg activity actually reduces in these children and adolescents with neurological gait disorders due to the DT exergame, although the original purpose of exergames was to increase motivation and active participation (see introduction). Despite that most muscles show smaller coefficients during stance in the DT condition, muscle activity during DT game playing was equal to or even higher than during regular walking in the Lokomat without the exergame. For example, amplitudes of the BF muscle during swing and stance as well as the GL muscle during stance were significantly higher (0.001 ≤ *p*-values ≤0.050) during the DT game condition. Thus, in line with earlier studies [5, 7], when children play such a demanding game, it results in a more active participation compared to normal walking in the DGO.

Another hypothesis was that not just the amount of leg activity would change but also the EMG-patterns. However, these patterns correlated high between the two game conditions. The DT condition apparently did not primarily influence the muscle activation pattern, and we had to reject that part of our second hypothesis. Interestingly, the mechanisms that made the walking look like compensatory for the therapist did not influence the activity patterns of the measured muscles. Hence, although the participants were distracted from their walking and somehow showed a reduced muscle activation in their legs, the patterns in the four measured muscles did not change. These mostly similar patterns might also be the result of the still accurately guided leg movements through the driven gait orthoses. Perhaps, but this remains speculative, different activations in other muscle groups accompanied the changed walking pattern in the patients showing compensatory strategies.

### Clinical implications

One of the important decisions a Lokomat therapist needs to do is to choose the right therapeutic inputs for each patient individually to attain the therapeutic goals. Our results show that this exergame might not be appropriate for all patients during robot-assisted gait therapy. We suggest that patients with higher motor abilities according to the FAC or WeeFIM mobility (but with poorer walking performance according to the GMFCS) might profit from a combination of walking in a DGO with an additional motor-cognitive task, because these patients can train with a physiological walking pattern, while they are still being challenged by the additional DT.

Furthermore, since the game scores do not correspond well to a physiological performance like in the earlier study of Labruyère et al. [17] a therapist cannot rely on these scores but has to carefully observe the child during the training. Furthermore, as shown in Schuler et al. [7], the inputs of a therapist additionally to walking in a DGO while game playing led to higher motor output. Such an approach of therapeutic advice during the DT game condition might also have a positive influence on the muscle activities and the walking performance when we would have applied it during this DT exergame. For example, participants ID3, 7, 8, 9 and 13, who walked with compensatory movements, might have profited from additional therapeutic inputs regarding their walking performance and all might have profited concerning the active participation of their legs during walking in the Lokomat and playing our new exergame. Moreover, in younger children, just implementing a game would not be enough to motivate the child (see patient ID14). We had to exclude this child (he was with 6.9 years old one of the youngest children) because he did not understand the meaning of the game without continuous inputs of the therapist. The influence of age in our study though was probably small, as age could not discriminate well between physiological or compensatory leg movements in the game conditions (DT game condition: AUC 0.32, *p* = 0.239; no DT game condition: AUC 0.11, *p* = 0.193).

Besides the age, also gait maturation could have influenced the walking performance of this demanding task. However, Sutherland [33] concluded that the gait pattern stabilizes between 3.5 to 4 years in healthy children and most of the remaining changes during the later years can be explained by the growth. Further studies which examined gait development in children found adult-like kinetic patterns in children of 5 years [34] and comparable gait kinetics and kinematics to adults at the age of 7 according to the hip and knee [35]. However, information on the development and maturation of more elaborate attention demanding gait tasks, as investigated in our study, are rare and difficult to compare to our DT task.

Related to the factor age and gait maturation, the developmental status of the brain should not be disregarded as well. The dorsolateral prefrontal cortex plays an major role in tasks where concentration is needed (e.g., complicated, novel or switch tasks) [36]. Additionally, when resources are limited, performing simultaneously a cognitive and motor task might be problematic [32]. Diamond (2002) [36] showed that the development of the dorsolateral prefrontal cortex already starts in early childhood with milestones between 3 and 6 and 7–11 years and continues until 25–30 years [36]. However, in children with cerebral palsy, their motor and cognitive development can be more or less affected depending on the extent of their brain lesions [37]. We also assume that motor and cognitive functions and their development in children with acquired brain injuries are more dependent on the localization and time point of this event. Unfortunately, we could not investigate into detail the neuropsychological executive functions in our participants. The results of the somewhat crude TONI4 indicate that this test is not a good discriminant factor for the performance of a DT robot-assisted gait therapy. We assume that this is because the TONI4 did not fully cover the cognitive dimensions that we consider relevant for this DT (i.e., attention and cognitive flexibility). Moreover, as we defined some prerequisites concerning the cognitive state in the inclusion criteria (sub point 8), we did not expect too large effects.

Finally, our results can be used by therapist to define clearer therapy criteria for patients who intend to train a physiological walking pattern with this DT exergame. While one could argue that we included a very heterogeneous group of participants, we consider the wide variety of diagnosis as beneficial for these therapeutic guidelines: the effects that we found seem rather robust throughout the patients that we train on a daily basis.

### Methodological considerations

One limitation of this study may be the non-blinded rating of the walking pattern since the Lokomat therapist knew which condition the patient was performing (test DT, test no DT, game DT, game no DT). Additionally, the therapist had no standardised checklist for rating the participants’ walking performances (i.e. physiological versus compensatory), which should be improved in a next study. However, the same therapist scored all participants, while she focused on step symmetry, compensatory strategies of game playing, and trunk control.

Although the number of participants was sufficient to determine differences between conditions, one should be cautious when interpreting the cut-off levels obtained from the ROC analyses.

Regarding the exergame, there were also some limitations. Firstly, children walked with a step-to-step variability, which resulted in inconsistent biofeedback values. Therefore, the standardised calculation of the threshold points for changing between the animals the wizard was riding on was difficult for some children. Secondly, most of the children were walking with one hand on the parallel bars and with the other one in the air while playing the DT exergame in the Lokomat. A few children could not use their other arm to support on the parallel bars. They generated a high muscle tone and arm flexor synergistic pattern during game playing which led to freehanded walking in the DGO. Walking free handed in a DGO is harder because of less support for trunk stability and resulted in compensatory walking performances (cf. Table 1 ID3 and ID7 with MACS level IV and a compensatory movement pattern during the DT condition).

A further limitation of the game was that the colour tracking of the magic wand functioned unreliable during a few measurements. This malfunction of tracking the wand reduced the children’s motivation to participate appropriately.

Finally, while the exergame during walking in a DGO induced overall higher EMG-amplitudes, for some patients with high muscle tone, a reduction of leg muscle activity could also be a positive issue. As mentioned in earlier studies from Schuler et al. 2011 [7], a higher muscle activity is not always desired or required in such patients. For us, it was therefore important to monitor and rate the quality of the gait pattern to identify compensatory walking strategies.

## Conclusion

We could show that this DT exergame increased active participation of children and adolescents with neurological gait disorders when undergoing a single session of robot-assisted walking and playing the exergame. Especially several performance measures could distinguish well between children who walked with a physiological versus a compensatory walking performance during the DT game condition. This differentiation might indicate that the game indeed trains a task that could be substantial for daily life ambulation. We also found specific effects on the EMG-amplitudes but no large effects on the EMG-patterns.

We assume that these results can be valuable for improving our understanding what such technologies can offer and in which patients a long-term DT robot-assisted gait therapy can be favourable and feasible. Additionally, it provides us with new therapy options to challenge also patients with higher motor and cognitive abilities in the Lokomat. We assume, in line with Liepelt et al. [38] that extensive DT therapy can result in an increased DT performance and that people can transfer it to new dual task situations. Further research is needed to investigate whether a long-term application of such a DT exergame during robot-assisted gait therapy indeed can increase the child’s everyday life ambulation faster and more efficient compared to no DT exergames.

## Abbreviations

AUC: Area under the curve from ROC analysis
BF: Biceps femoris muscle
DA: Deltoideus pars acromialis muscle
DGO: Driven gait orthosis
DT: Dual task
FAC: Functional Ambulation Category
Game DT: Game with dual task
Game nDT: Game without dual task
GL: Gastrocnemius caput lateralis muscle
GMFCS: Gross Motor Function Classification System
ICF: International Classification of Functioning Disability and Health
MACS: Manual Ability Classification System
MMT: Manual muscle test
nDT: No dual task
pw: Physiological walking
RAGT: Robot-assisted gait therapy
ROC analysis: Receiver operating characteristic analysis
SCALE mleg: Selective control of the lower extremity of the measured leg
TA: Tibialis anterior muscle
TONI4%ile Rank: Test Of nonverbal Intelligence fourth version, percentile rank score
VMO: Quadriceps femoris pars vastus medialis obliquus muscle
WeeFIM mob: Functional independence measure for children, subscale mobility
